# Prediction of dispositional dialectical thinking from resting‐state electroencephalography

**DOI:** 10.1002/brb3.2327

**Published:** 2021-08-22

**Authors:** Kun Huang, Dian Chen, Fei Wang, Lijian Yang

**Affiliations:** ^1^ Center for Statistical Science and Department of Industrial Engineering Tsinghua University Beijing China; ^2^ Department of Psychology School of Social Sciences Tsinghua University Beijing China; ^3^ Laboratory of Brain and Intelligence Tsinghua University Beijing China

**Keywords:** alpha wave, dialectical thinking, FDA, machine learning, resting‐EEG

## Abstract

This study aims to explore the possibility of predicting the dispositional level of dialectical thinking using resting‐state electroencephalography signals. Thirty‐four participants completed a self‐reported measure of dialectical thinking, and their resting‐state electroencephalography was recorded. After wave filtration and eye movement removal, time‐frequency electroencephalography signals were converted into four frequency domains: delta (1–4 Hz), theta (4–7 Hz), alpha (7–13 Hz), and beta (13–30 Hz). Functional principal component analysis with B‐spline approximation was then applied for feature reduction. Five machine learning methods (support vector regression, least absolute shrinkage and selection operator, K‐nearest neighbors, random forest, and gradient boosting decision tree) were applied to the reduced features for prediction. The model ensemble technique was used to create the best performing final model. The results showed that the alpha wave of the electroencephalography signal in the early period (12–15 s) contributed most to the prediction of dialectical thinking. With data‐driven electrode selection (FC1, FCz, Fz, FC3, Cz, AFz), the prediction model achieved an average coefficient of determination of 0.45 on 200 random test sets. Furthermore, a significant positive correlation was found between the alpha value of standardized low‐resolution electromagnetic tomography activity in the right dorsal anterior cingulate cortex and dialectical self‐scale score. The prefrontal and midline alpha oscillations of resting electroencephalography are good predictors of the dispositional level of dialectical thinking, possibly reflecting these brain structures’ involvement in dialectical thinking.

## Graphical abstract

This study aims to explore the possibility of predicting the dispositional level of dialectical thinking using resting‐state electroencephalography signals.The result shows that the prefrontal and midline alpha oscillations of resting electroencephalography are good predictors of the dispositional level of dialectical thinking.

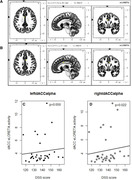

## 1 INTRODUCTION

We live in a world full of contradictions. One possible way to deal with conflicting information we face every day is *dialectical thinking*, which involves viewing the world through a dynamic and holistic lens and accepting and resolving the inevitable contradictions (Peng & Nisbett, 1999).

Dialectical thinking has been considered by developmental psychologists as a sophisticated form of thinking that emerges in the final stage of cognitive development (Basseches, 1980; Piaget, 1974; Riegel, 1973). Later cross‐cultural research demonstrated that dialectical thinking is especially prevalent in East Asian cultures with three central principles: (i) the principle of change, which states that everything is constantly changing; (ii) the principle of contradiction, which states that contradiction exists everywhere and even coexists within the same thing; and (iii) the principle of relationships or holism, which states everything is connected (Peng & Nisbett, 1999). Although, some individuals from Western cultures may also use it regularly.

Dialectical thinking has overarching impact on cognitive processes as various levels (Spencer‐Rodgers & Peng, 2018; Spencer‐Rodgers et al., 2010), and an inquiry into the underlying brain mechanisms may deepen our understanding of how dialectical thinking exerts its effects on human cognition. Among the three principles of dialecticism, the principle of contradiction has received a lot of attention. Functional magnetic resonance imaging (fMRI) studies have revealed that the dorsal anterior cingulate cortex (dACC) plays a key role in the monitoring and resolution of conflicting information (Botvinick et al., 2004; Cachia et al., 2017; Carter & van Veen, 2007; Wang et al., 2016). Furthermore, within a broader framework of executive functions and cognitive control (Cohen, 2017; Diamond, 2013), dealing with contradiction requires a heightened level of executive control. Accordingly, neuroimaging studies have found that the processing of contradiction involves attentional control network regions, such as the dorsolateral prefrontal cortex (DLPFC), (Botvinick et al., 2004; Carter & van Veen, 2007; Egner, 2007). These fMRI findings were further supported by brain lesion studies, which have also shown that a common consequence of dACC injuries is the inability to reliably eliminate conflict‐driven behaviors (Mansouri et al., 2017), and the DLPFC is also associated with conflict processing (Botvinick et al., 2004; Egner, 2007).

Another line of research has utilized the superior temporal resolution of electroencephalography (EEG) to examine the temporal features of the brain mechanisms underlying conflict processing. Studies that employed paradigms such as the Stroop task (Badzakova‐Trajkov et al., 2009; Chuderski et al., 2016), go/no‐go task (Kostyrka‐Allchorne et al., 2019), flanker conflict task (Kanske & Kotz, 2010; Tillman & Wiens, 2011), Simon task (De Ridder et al., 2011; Galashan et al., 2008), and speeded response task (Sokhadze et al., 2008) suggest that the occurrence of cognitive conflict is often associated with an increased N2 and N450 component. Furthermore, several studies have demonstrated that EEG features, such as EEG rhythmic activity (e.g., delta, theta, alpha, and beta) change as a function of contradiction processing (Almabruk et al., 2016; Moore et al., 2012; Nakao et al., 2013; Pornpattananangkul et al., 2019). Delta rhythmic activity is related to behavioral inhibition (Kamarajan et al., 2004; Knyazev, 2007; Putman, 2011), theta‐band (4−8 Hz) rhythmic activity supposedly reflects neural mechanisms of conflict detection (Cavanagh & Frank, 2014), alpha rhythmic activity is related to conflict processing (Capuron et al., 2005; Jiang et al., 2015; Wacker et al., 2010), beta rhythmic activity plays a role in conflict detection (Chen et al., 2020).

For example, in the occupation choice task, the high‐contradiction group had greater delta and theta power in the N2 amplitude in the frontocentral region than the low‐conflict group (Nakao et al., 2013). Additionally, in the signal stop task, the frequency band from 1 to 7 Hz (i.e., delta and theta range) is induced at 800 ms (Andersen et al., 2009; Moore et al., 2006; Savostyanov et al., 2009). In contrast, the amount of conflict was associated with alpha and beta frequencies in the left occipitotemporal regions (Nakao et al., 2013).

Even though these studies provide valuable insight into the question of how the brain processes conflicting information, direct investigations into the neural bases of dialectical thinking have been scarce. To the best of our knowledge, only one recent fMRI study directly examined the effect of dispositional dialectical thinking on the brain. Wang et al. (2016) used a modified self‐reference paradigm to present participants with contradictory or noncontradictory personality adjective pairs and recorded their brain activities when making self or other judgments. They found that the level of dialectical thinking positively correlated with the dACC's involvement in the processing of self‐relevant contradictions. Based on this finding, they suggest that the critical difference between dialectical and nondialectic thinkers is how likely they are to utilize the dACC to modulate other regions’ activities.

While Wang et al. (2016) provided initial evidence regarding the neural basis of dialectical thinking, there are still issues to be clarified. First, their study was exclusive to the domain of the self, and it is still not clear whether dispositional dialectical thinking may also manifest in the brain's stable and task‐free activity patterns, such as in the resting state. Intriguingly, the dACC is a part of the salience network (SN), which governs the allocation of attention to stimuli based on their subjective salience (Seeley et al., 2007; Sridharan et al., 2008). SN has a key role in switching between the default mode network (Buckner et al., 2008) and executive control network (Osaka et al., 2004), and these networks interact with each other even in the resting‐state. Therefore, it is worthy to examine the link between dialectical thinking and resting‐state brain activity. Second, the fMRI technique they used, while advantageous in localizing the involved brain regions, cannot portray the finer temporal features of the neural mechanisms. Finally, their study used a correlational approach by associating certain brain features with a behavioral index, which can be supplemented by a predictive approach that combines neural data and machine learning (ML) algorithms to achieve individualized predictions and uses cross‐validation techniques to ensure out‐of‐sample generalizability (Dubois & Adolphs, 2016).

In the current study, we aim to explore the possibility of predicting the level of dispositional dialectical thinking via resting‐state EEG features. To achieve this goal, we need to deal with the “curse of dimensionality,” that is, the brain voltage captured by EEG is usually measured thousands of times while the number of experimental subjects is small, posing huge challenges to traditional data analysis methods. Traditional EEG data analysis methods usually include manually extracting physiological features (such as frequency, spectral power, etc.) from EEG signals. A common problem of this manual feature selection strategy is choosing the type of features. EEG data contain a complex structure that makes it difficult to filter useful information simply via predefined features. Therefore, a data‐driven prediction method capable of auto feature selection from the data while keeping as much information as possible is preferable.

In such situations, functional data analysis (FDA) provides a useful statistical approach for dealing with this problem. Through smoothing and decomposing, FDA eliminates data noise and extracts principal components representing most of the information from data. Recently, Zhang et al. (2020) applied the FDA method to predict working memory ability based on EEG and achieved great accuracy, demonstrating the feasibility of this approach.

Here, we first applied FDA to extract EEG features using R (Ripley, 2001) software to provide a feature representation of individual subjects. We then applied a set of ML methods to predict participants’ scores on the Dialectical Self Scale (DSS; Spencer‐Rodgers et al., 2004), a widely used self‐reported measure of dialectical thinking. Third, we performed a randomness test on our result to distinguish it from random noise. Finally, using eLORETA for source analyses, our specific aim was to test for the relevance of EEG‐based resting state activity in dACC and DLPFC for dialectical thinking. Based on previous findings (Botvinick et al., 2004; Carter & van Veen, 2007; Egner, 2007; Wang et al., 2016), we hypothesized that the degree of EEG‐based resting state activity in the dACC (as measured using eLORETA values) is related to the degree of dialectical thinking.

## 2 MATERIAL AND METHODS

### 2.1 Participants

A total of 37 Chinese‐speaking participants were recruited from Tsinghua University, China. Participants were healthy, had no history of neurological disorder, normal or corrected to normal vision, and all were right‐handed. Three participants had to be excluded from further analysis because of excessive EEG artifacts, leaving a sample of 34 participants (18 women, 18−30 years old, mean age = 23 years, standard deviation = 3.1). Informed consent was obtained from all participants prior to the experiment according to procedures approved by the Ethics Committee of the Department of Psychology, Tsinghua University and all methods were performed in accordance with the relevant guidelines and regulations.

### 2.2 Measure of dispositional dialectical thinking

Dispositional dialectical thinking was assessed using the Dialectical Self Scale (DSS)(Spencer‐Rodgers et al., 2004), with the 32 items rated on 1 (strongly disagree) to 7 (strongly agree) scale. Sample items include “I often find that things will contradict each other,” “My world is full of contradictions that cannot be resolved,” and “When two sides disagree, the truth is always somewhere in the middle.” In the cross‐cultural psychological literature, DSS has been used widely and has shown adequate reliability and validity have been confirmed in pieces of literature (Hamamura et al., 2008; Hui et al., 2009; Spencer‐Rodgers et al., 2009). In the current study, the Cronbach's alpha was .74, which was comparable to previous studies (e.g. .74 for Chinese participants in Spencer‐Rodgers et al., 2009).

### 2.3 EEG recording

Five minutes of open‐eye resting‐state EEG data were recorded using an EEG amplifier and Ag/CI electrodes through a 64‐channel cap (according to the International 10/20 system) referenced to the left mastoid TP9. The data were sampled at 500 Hz. The impedance of each electrode was kept under 5 kΩ. The EEG data preprocessing was performed using the Fieldtrip (Oostenveld et al., 2011) toolbox for MATLAB 2019b.

### 2.4 Data analysis

#### 2.4.1 Data preprocessing

The EEG data collected clearly contained machine noises. The frequency of machine noise is usually assumed to be above 40 Hz. In the data preprocessing step, EEG signals were filtered by Finite Impulse Response (FIR) to be between 1 and 40 Hz. While the signal was collected during the eyes open state, independent component analysis (ICA) was performed on the EEG data to remove the eye‐related component, which is assumed to be a major disturbance to the signal.

The EEG series at the beginning time is considered to be noisy since the participants might not have been in the required state. Since it is hard to decide a subject‐specific noisy period for each subject, the first 2.5 s, which is considered to be long enough to cover noisy periods for all subjects, is excluded automatically. Another reason for excluding the same EEG length for all subjects is so that the data for analysis under the same condition, which is the requirement of the FDA theory. Similarly, signals at the end 45.5 s were excluded (too noisy because the participants might have failed to stay still after having been sitting too long). An EEG series with 252 s of data was obtained for each subject's electrode.

The collected EEG signals were analyzed in two different ways. First, the whole signal series was considered as a predictor for the DSS score. In addition, the whole EEG series was segmented into consecutive disjointed pieces of 3 s (or 1500 measurements). This segmentation leads to 84 periods in total in chronological order with each period treated as a separate predictor, the *p*th period after segmentation, 0 ≤ *p* ≤ 83, are consecutive and disjointed, while the 84th period denotes the whole signal series. Following the convention of FDA theory, the original measurement index was rescaled by$\;N_{p}$, the number of measurements in a single period, so that the time domain is [0,1] for simplicity of notations and computation. For each period, electrode, and subject, the corresponding EEG series was also centralized so that the sum of each EEG series’ voltage across the time is equal to zero. The purpose of the centralization is to rescale different EEG series and is important in the subsequent FDA procedure. After centralization, FIR filter (with hamming window and the length of the filter is 400) was applied to the signal and four types of waves are extracted: delta wave (1−4 Hz), theta wave (4−7 Hz), alpha wave (7−13 Hz), and beta wave (13−30 Hz).

The EEG data with all the above preprocessing are denoted as $Y_{w,p,l,i}\left(\frac{j}{N_{p}}\right)$, where 1≤w≤4 denotes four types of waves, 0≤p≤84 different periods, 1≤l≤63 different electrodes, 1≤i≤34 experimental subjects, and $1\;\leq \;j\;\leq \;N_{p}$ measurement time points within each period.

#### 2.4.2 Functional data analysis

FDA provides a useful statistical approach for processing high frequency signal data (Ramsay & Silverman, 1997). FDA uses some basis functions to approximate the underlying continuous process from discrete observations. The basis functions can be predetermined (e.g., Fourier basis, B‐splines) or data‐driven. The introduction of the basis function is a key step in dimension reduction, where an infinite‐dimensional function space is reduced to finite vector space. The number of reduced vector dimensions is a hyperparameter and can be determined according to the signal characteristics. Once the basis functions are well estimated, the signal can be approximated using a linear combination of these basis functions, with the linear coefficients representing the underlying characteristics of the signal data.

Another advantage of the FDA approach is its theoretical support. The EEG data collected is usually contaminated with certain artifacts (like muscle artifacts, electrocardiogram, etc.) which, in general, are difficult to handle it. Fortunately, the FDA theory shows that under certain conditions, the noise contaminating the EEG signals can be removed in an asymptotic sense with the help of B‐spline estimator(Wang et al., 2020). So, in this paper, we used the FDA approach as a tool to remove artifacts and obtain useful information from the noisy EEG data.

For every possible wave, *w*; period, *p*; and electrode, *l*; the EEG series $Y_{w,p,l,i}\left(\frac{j}{N_{p}}\right)$ of the *i*th subject is decomposed as

$$\begin{array}{ccc} Y_{w,p,l,i}\;\left(\frac{j}{N_{p}}\right) & = & m_{w,p,l}\;\left(\frac{j}{N_{p}}\right)+\sum_{k\;=\;1}^{\infty }\xi_{w,p,l,i,k}\phi_{w,p,l,k}\left(\frac{j}{N_{p}}\right)\\ & & +\;\varepsilon_{w,p,l,i}\left(\frac{j}{N_{p}}\right),\;1\leq j\leq N_{p}, \end{array}$$

where $m_{w,p,l}\left(\cdot \right)$ is the common mean function of all subjects, $\phi_{w,p,l,k}\;\left(\cdot \right)$ is the *k*th eigenfunction, and $\xi_{w,p,l,i,k}$ is is the functional principal component score for the *i*th subject, which accounts for intersubject variation in the signal. $\varepsilon_{w,p,l,i}\left(\frac{j}{N_{p}}\right)$ represents measurement errors. The key step of dimension reduction is to estimate $\left\{\xi_{w,p,l,i,k}\right\}$ k up to κ, a hyperparameter that is the smallest integer such that the largest κ eigenvalues amount to at least 95% of the sum of all eigenvalues. The basis function, $\phi_{w,p,l,k}\;\left(\cdot \right)$, and mean function, $m_{w,p,l}\left(\cdot \right)$, are approximated by B‐splines. The order of the spline basis is chosen to be two (linear basis) and the number of spline bases is$\;[c\;\times \;N_{p}^{1/4}\;\times \;log\left(N_{p}\right)]$, where *c* = 1.4. The estimation procedure is introduced in Appendix A.

#### 2.4.3 Machine learning approach

**FIGURE 1:**
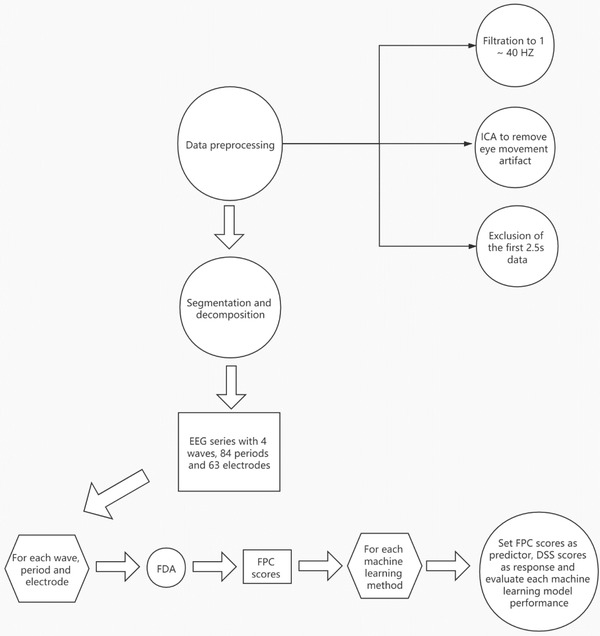
Flow chart of data analysis

After the FDA approach, the data‐driven features ${\left\{\xi_{w,p,l,i,k}\right\}}_{1\leq i\leq 34,1\leq k\leq \kappa }$, which are considered to represent most of information from EEG signal but has implicit physiological meaning, are obtained for each set of wave, period, and electrode, ${\left\{w,p,l\right\}}_{1\leq w\leq 4,0\leq p\leq 84,1\leq l\leq 63}$. The integer κ here is the smallest number by which the data‐driven features ${\left\{\xi_{w,p,l,i,k}\right\}}_{1\leq i\leq 34,1\leq k\leq \kappa }$ is able to account for 95% variation of the data and more detail is discussed in Appendix A. For each wave *w*, period *p*, and electrode l, five machine learning methods, denoted as *m*, were applied to features ${\left\{\xi_{w,p,l,i,k}\right\}}_{1\leq i\leq 34,1\leq k\leq \kappa },$ which has dimension 34×κ, to predict the DSS score. The above procedure resulted totally 4×85×63×5=107,100 models. To evaluate each model's performance, 23 subjects were randomly sampled 200 times as the training set and the rest as the testing set. At each sampling s, the model was fit on the training set, and the coefficient of determination $R_{w,p,l,m,s}^{2}$ is computed for the testing set. We took an average of these 200 samplings’ $R_{w,p,l,m,s}^{2}\;$as a performance evaluation of the model. The definition of $R_{w,p,l,m,s}^{2}$ is

$$\;R_{w,p,l,m,s}^{2}=\;1-\frac{\sum_{i\;=\;1}^{n_{t}}{\left(S_{w,p,l,m,s,i}-{\hat{S}}_{w,p,l,m,s,i}\right)}^{2}}{\sum_{i\;=\;1}^{n_{t}}{\left(S_{w,p,l,m,s,i}-{\bar{S}}_{w,p,l,m,s}\right)}^{2}},$$

where $S_{w,p,l,m,s,i}$ is the DSS score of subject *i* from the testing set,$\;{\hat{S}}_{w,p,l,m,s,i}$ is the predicted score from model, $n_{t}$ is the sample size of the testing set, and ${\bar{S}}_{w,p,l,m,s}=n_{t}^{-1}\;\sum_{i\;=\;1}^{n_{t}}S_{w,p,l,m,s,i}$. By the definition of $R_{w,p,l,m,s}^{2}$, the numerator denotes the sum of squared errors of model m, while the denominator denotes the sum of squared errors from the baseline model, where all the subjects’ scores are predicted by their mean. When $R_{w,p,l,m,s}^{2}$ is negative, the model is considered worse than the baseline model and the model is useful if $R_{w,p,l,m,s}^{2}>0$. To be clearer, each step of the data analysis is shown in Figure 1.

The five machine learning methods include support vector regression (SVR)(Drucker et al., 1997), least absolute shrinkage and selection operator (LASSO)(Tibshirani, 1996), K‐nearest neighbors (KNN)(Altman, 1992), random forest (RF)(Ho, 1995), and gradient boosting decision tree (GBDT)(Friedman, 2001). SVR is similar to support vector machine (SVM), which attempts to maximize the margins of the support vector plane and is a popular classification method. The SVR model is used in (Al Zoubi et al., 2018) to predict age from EEG signal and is suitable for our problem. A radial basis kernel is used in the SVR model. LASSO is a linear regression model with absolute error regularization and is popular for feature selection due to its sparse regression result. Linear regression is the simplest approach for prediction or inference. Since the number of subjects is rather small, regularization is important in the fitting model and that is why LASSO is adopted. The regularization coefficient of the LASSO model was set to 1. To be more flexible and not restricted in linear relationship, KNN regression is applied due to its simple assumptions and comprehensibility. The K was defined as 3 in KNN model. Besides the simple learning algorithm, some ensemble learning approaches, including bagging and boosting algorithms, were taken to compare their performance in predicting DSS problems. RF is a type of bagging algorithm and is flexible in dealing with high‐dimensional data. The randomness of the feature selection of RF can adjust for the noisy EEG data and make the prediction result more stable. The number of random trees in the RF was chosen to be 15. GBDT is a boosting algorithm designed to iteratively remove prediction bias. Wu et al. (2017) use GBDT to evaluate emotion from EEG signal and achieve good performance. It is expected to perform well in our problem. The number of boosting stages was 20 and the learning rate was 1. From a simple linear relationship to nonlinear representation, from a single model (SVR, LASSO, and KNN) to the model ensemble (RF and GBDT), these five machine learning methods cover most mainstream machine learning methods and are capable of adapting to complex situations.

Finally, for every wave (*w*), period (*p*), electrode (*l*), and machine learning method (*m*), the ${\bar{R}}^{2}$, averaged coefficient of determination among 200 samplings, is computed. We use subscript to distinguish different ${\bar{R}}^{2}$ from different settings and denoted them as$\;{\bar{R}}_{w,p,l,m}^{2}$. Because ${\bar{R}}_{w,p,l,m}^{2}$ is actually a random variable indicating whether the model is useful, a threshold of 0.1 was chosen, above which the prediction model was considered to be helpful (because the estimated standard error of ${\bar{R}}_{w,p,l,m}^{2}$ is much less than 0.1, this threshold is considered to be sufficient for detecting useful models).

To obtain a more powerful prediction model, we used the model ensemble technique to develop a useful model. The results are shown in the next section.

#### 2.4.4 Randomness hypothesis test

In statistics, performing multiple hypothesis tests simultaneously electrodes to a multiple testing problem (Rupert, 2012). This is relevant to the present study because $4\times 84\times 63\;=\;21,168\;{\bar{R}}_{w,p,l,m}^{2}$ values were calculated for each machine learning method in the second part of the analysis. Are these ${\bar{R}}_{w,p,l,m}^{2}>0.1$ results caused by the inherent randomness of the multiple calculations? Some hypothesis tests determining the randomness of the result were conducted. For a particular method, *m*, the null hypothesis is ${\bar{R}}_{w,p,l,m}^{2}$ is an identical individually independent random variable among all sets of ${\left\{w,p,l\right\}}_{1\leq w\leq 4,0\leq p\leq 83,1\leq l\leq 63}$. Under the null hypothesis, the number of results with ${\bar{R}}_{w,p,l,m}^{2}>0.1$ should be distributed uniformly across the four types of waves. Let p be the probability of ${\bar{R}}_{w,p,l,m}^{2}>0.1$. On the other hand, suppose another situation where the probability of ${\bar{R}}_{w,p,l,m}^{2}>0.1$ varies across different waves, which is denoted as $p_{w}=\;P\left({\bar{R}}_{w,p,l,m}^{2}>0.1\right)$. The likelihood ratio test was then performed to decide whether the null hypothesis should be rejected.

The reason waves other than periods were chosen to perform the test is that there are a total of 84 periods and values of ${\bar{R}}_{w,p,l,m}^{2}>0.1$ may have a sparse distribution over these many periods, which will cause an infinite result in the maximum likelihood estimation. The same logic applies to the electrodes. A particular method was fixed before the test because different methods with the same wave, period, and electrode will cause correlated results, which violates the independent hypothesis.

#### 2.4.5 Exact low‐resolution brain electromagnetic tomography analysis

Low‐resolution brain electromagnetic tomography (LORETA) is a source‐analysis technique designed to estimate the location and activity of neural generators that cause EEG activity in the scalp. It was developed by the KEY Institute of Brain‐Mind Research at the University of Zurich (Pascual‐Marqui et al., 1994) to calculate the three‐dimensional distribution of neural current density sources in the brain. Two improvements to this method have been published, standardized low‐resolution electromagnetic tomography (sLORETA), which uses standardized current density to calculate intracerebral generators (Pascual‐Marqui, 2002), and exact low‐resolution electromagnetic tomography (eLORETA), which has no need for standardized correct positioning (Pascual‐Marqui, 2007) and is a more precise locator of possible current density sources.

The current eLORETA approach uses a real head model (Fuchs et al., 2002) and electrode coordinates (Tsuzuki et al., 2007). The steps to calculate eLORETA values are as follows: (1) electrode names to coordinates, (2) electrode coordinates to transformation matrix, (3) EEGs to cross spectrum, (4) cross spectra to sLORETA, (5) ROI creation (the dACC and DLPFC ROIs were defined using all voxels within 5 mm of the following seeds [Table 1] [Montreal Neurological Institute coordinates]) (Damasio, 1996; De Ridder et al., 2011; Song et al., 2014), and (6) sLORETA to ROIs.

**TABLE 1:** Seeds used for ROI definition

	*X*	*Y*	*Z*
Left dACC	−8	2	36
Right dACC	7	1	36
Left DLPFC	−48	33	38
Right DLPFC	53	17	50

Abbreviations: dACC, dorsal anterior cingulate cortex; DLPFC, dorsolateral prefrontal cortex.

#### 2.4.6 Statistical correlations

For each resting‐state oscillation (delta, theta, alpha, beta) and DSS score, one‐tailed Spearman's correlation tests were performed. To account for the multiple correlation tests performed in this study, Bonferroni correction was applied in R packages to *p* values of interests.

### 2.5 Result

### 2.6 Behavioral data result

**FIGURE 2:**
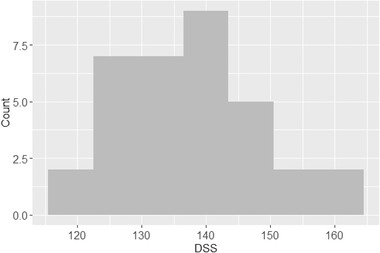
Histogram of the DSS score

The mean DSS score was 137.65, with a standard deviation of 10.71. Figure 2 shows the histogram of the DSS scores. It can be seen the range of the DSS score is from 115 to 165. Most of the scores are about 140.

### 2.7 Whole EEG results (*p *= 84)

The results show that the whole EEG series (*p* = 84) is not a good predictor of the DSS score. None of the models achieved an ${\bar{R}}^{2}$ higher than 0.1. The results with ${\bar{R}}^{2}$ higher than 0 are displayed in Table 2. Since the ${\bar{R}}^{2}$ calculated here is a random variable, there is little confidence about whether these settings are really helpful in predicting the DSS score. The results posted here serve as a reference for future studies in this field.

**TABLE 2:** Results of models with ${\bar{R}}^{2}>0$

Wave	Period	Electrode	Method	*R* ^2^
Alpha	84	FCz	KNN	0.058
Alpha	84	Cz	RF	0.053
Alpha	84	Cz	KNN	0.047
Beta	84	F5	RF	0.027
Alpha	84	FCz	GBDT	0.025
Alpha	84	FCz	RF	0.007

Abbreviations: GBDT, gradient boosting decision tree; KNN, K‐nearest neighbors; LASSO, least absolute shrinkage and selection operator; RF, random forest.

### 2.8 Segmental EEG results (0 ≤ *p* ≤ 83)

Among the segmented periods (0 ≤ *p* ≤ 83), there were a total of 35 settings that achieved an ${\bar{R}}^{2}$ > 0.1. Table 3 lists the information of these settings.

**TABLE 3:** Model results with ${\bar{R}}^{2}$ > 0.1

Wave	Period	Electrode	Method	*R* ^2^
Alpha	4	FC1	LASSO	0.376
Alpha	4	FCz	LASSO	0.350
Alpha	27	CP2	LASSO	0.326
Beta	6	FC1	LASSO	0.292
Alpha	5	C5	LASSO	0.244
Alpha	27	P2	LASSO	0.237
Theta	42	AF3	LASSO	0.230
Alpha	8	FT7	LASSO	0.207
Alpha	4	FZ	LASSO	0.202
Theta	69	CP4	LASSO	0.192
Alpha	27	P2	RF	0.188
Theta	11	AF7	LASSO	0.183
Beta	65	FT8	LASSO	0.179
Alpha	37	CP6	LASSO	0.179
Alpha	82	P5	LASSO	0.165
Alpha	25	FZ	LASSO	0.162
Alpha	4	FC3	LASSO	0.158
Alpha	4	Cz	LASSO	0.152
Alpha	8	FC3	GBDT	0.148
Alpha	63	C2	LASSO	0.142
Beta	10	TP8	RF	0.136
Alpha	4	AFz	LASSO	0.135
Theta	14	FP1	LASSO	0.125
Delta	45	T7	GBDT	0.123
Alpha	4	Cz	RF	0.121
Delta	71	P7	LASSO	0.120
Delta	82	T7	KNN	0.120
Alpha	32	FCz	RF	0.120
Alpha	43	CP1	LASSO	0.118
Theta	69	CP2	LASSO	0.114
Delta	47	FC2	RF	0.112
Theta	13	O2	GBDT	0.108
Theta	47	P1	LASSO	0.108
Theta	47	PO4	RF	0.103
Delta	80	FZ	LASSO	0.101

Abbreviations: GBDT, gradient boosting decision tree; KNN, K‐nearest neighbors; LASSO, least absolute shrinkage and selection operator; RF, random forest.

**FIGURE 3:**
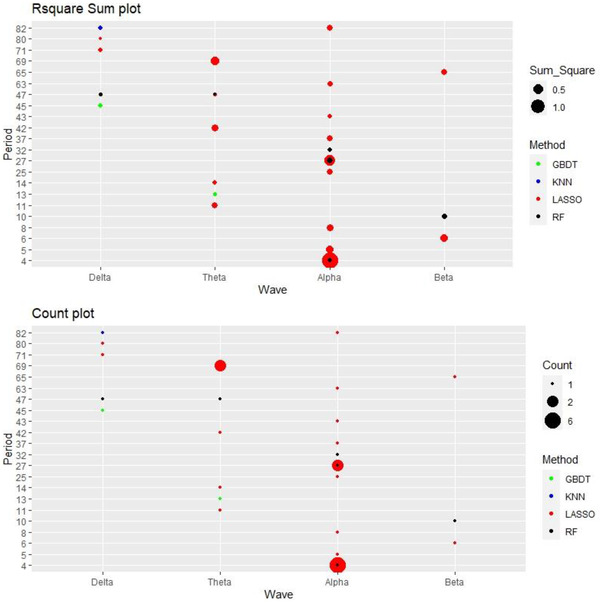
Performance of different settings. Abbreviations: LASSO, least absolute shrinkage and selection operator; KNN, K‐nearest neighbors; RF, random forest; GBDT, gradient boosting decision tree

Two kinds of measurements were used to assess the predictive value of different methods, waves, and periods. First, the frequency of each setting was calculated and is shown in Table 3. A higher frequency indicates a higher predictive ability. Second, all ${\bar{R}}^{2}$ values calculated under each setting were summed, and the summation was used to represent their predictive value. Figure 3 shows these two measurements for different settings. Different colors denote different methods. There is no SVR method in Figure 3 because no ${\bar{R}}^{2}>0.1$ results were obtained using the SVR method. The size of points denotes the value of the summed ${\bar{R}}^{2}$ values or count. Periods and waves with no values of ${\bar{R}}^{2}>0.1$ are not presented in the Figure 3. These two measurements are used to compare the performance of different settings in the following subsections.

#### 2.8.1 Best *R* ^2^

**FIGURE 4:**
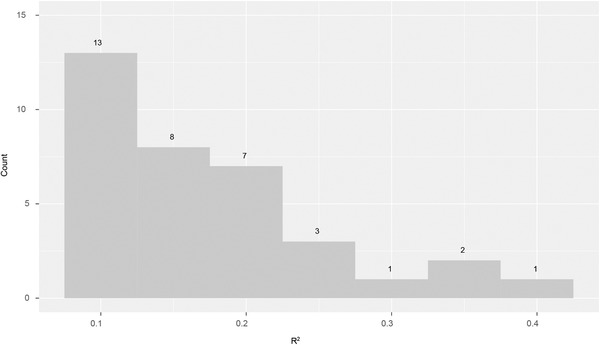
Histogram of values of ${\bar{R}}^{2}$ *>* 0.1

As shown in Table 3, the highest ${\bar{R}}^{2}$ was 0.376, obtained with wave alpha, period 4, and electrode FC1, which suggests a strong relationship between these predictors and DSS score. Figure 4 shows a histogram of values of ${\bar{R}}^{2}$ > 0.1. While there are a few values of ${\bar{R}}^{2}$ ≥ 0.3, most ${\bar{R}}^{2}$ values are less than 0.2.

#### 2.8.2 Different machine learning method results

**FIGURE 5:**
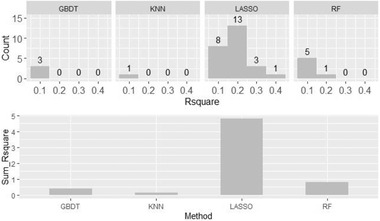
Result of different methods with a value of ${\bar{R}}^{2}>0.1$. Abbreviations: LASSO, least absolute shrinkage and selection operator; KNN, K‐nearest neighbors; RF, random forest; GBDT, gradient boosting decision tree

Among these 35 helpful models, the performance of different machine learning methods was examined. As shown in Figure 3, most dots are red in color, indicating that the LASSO method plays an important role in predicting the DSS score. Figure 5 shows the histogram and sum of ${\bar{R}}^{2}$ grouped by different machine learning methods. Because there was no SVR model with a value of ${\bar{R}}^{2}$ > 0.1, the SVR method is not shown in the figure 5. With more than half of the results belonging to the LASSO model, the LASSO method clearly outperformed the other four methods. Meanwhile, the ${\bar{R}}^{2}$ values obtained using the other four methods were quite small and less than 0.2 in almost all the cases, while the sum of ${\bar{R}}^{2}$ values obtained using the LASSO method is much larger than that for the other methods. LASSO is thus considered to be the most suitable method for dealing with the DSS prediction problem with the help of FPCA in this study.

#### 2.8.3 Different period results

**FIGURE 6:**
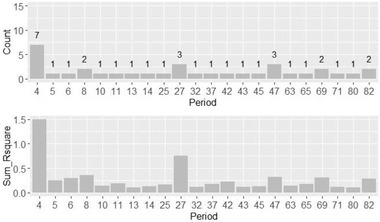
Result of different periods with ${\bar{R}}^{2}$ *>* 0.1

Since there were 84 periods, only periods with a value of ${\bar{R}}^{2}$> 0.1 are displayed for the simplicity of visualization. Figure 6 shows the count and ${\bar{R}}^{2}$ sum for each period. It can be seen from the Figure 6 that period 4 plays an important role in the prediction accuracy. In total, period 4 appears seven times, while most other periods appear only one time at most.

Meanwhile, the sum of ${\bar{R}}^{2}$ values for period 4 is almost 1.5, which far exceeds the value for other periods. On the other hand, periods 4, 5, and 6 are the only three consecutive periods that appear on the histogram. Although some other periods also have values of ${\bar{R}}^{2}$ > 0.1, there is no obvious evidence regarding how they correlate. The time represented by periods 4, 5, and 6 is assumed to be a period when the participants were still new to the experimental environment and gradually adapting to it. This result suggests that the EEG signal at the very beginning of the experimental period may relate to the participant's DSS score.

#### 2.8.4 Different waves results

**FIGURE 7:**
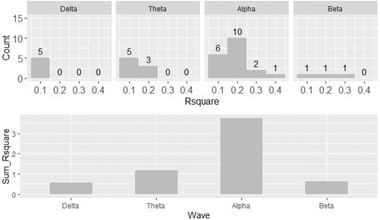
Result of different waves with ${\bar{R}}^{2}$ *>* 0.1

Among the results with ${\bar{R}}^{2}$ > 0.1, the ability of different waves to predict the DSS score was compared. As shown in Figure 3, the alpha wave appears the most times among the results with ${\bar{R}}^{2}$> 0.1 as well as with the highest sum of ${\bar{R}}^{2}$, which suggests that the alpha wave has the best predictive accuracy. Figure 7 shows the distribution of ${\bar{R}}^{2}$grouped by wave. The alpha wave is much more important in predicting the DSS score than the other three waves. The delta wave's summed ${\bar{R}}^{2}$ was rather low, while the beta wave produced the least valuable models. The theta wave served as the second most important predictor but still did not have a sufficiently high ${\bar{R}}^{2}$.

#### 2.8.5 Randomness test result

Since the LASSO method far exceeded the other methods, the likelihood ratio test was only performed for the LASSO results. The test statistic was computed to be 16.68, with a chi‐square distribution with three degrees of freedom, which suggests a *p* value of 0.0008. In addition, the identical individually independent distribution of ${\bar{R}}^{2}$ of the other three waves was also tested (δ wave, θ wave, and β wave) and the test statistic was 2.97 with a chi‐square distribution of two degrees of freedom, which suggests a *p* value of 0.23.

Thus, based on the randomness test result, we concluded that these ${\bar{R}}^{2}$ > 0.1 results are not simply caused by randomness. The alpha wave shows a significant difference from the other three waves, while the null hypothesis regarding the randomness of the δ, θ, and β wave results was not rejected.

#### 2.8.6 Model ensemble accuracy

To further improve model accuracy, models using different predictors were combined to reduce the prediction error. The results shown above indicate the strong importance of the alpha wave, 4th period, and LASSO method. It can be seen from Table 3 that these settings used data from six electrodes, namely FC1, FCz, Fz, FC3, Cz, and AFz, with ${\bar{R}}^{2}$ 0.376, 0.350, 0.202, 0.158, 0.152, and 0.135 respectively. Thus, the model obtained with these electrodes under the best settings was used to form our final predictive model:

$$\;M_{ensemble}=\frac{1}{6}\;\left(M_{FC1}+M_{FCz}+M_{Fz}+M_{FC3}+M_{Cz}+M_{AFz}\right),$$

where $M_{lead}$ denotes the model predicting DSS score using that particular electrode as well as the alpha wave, 4th period, and LASSO method. The model ensemble's average $R^{2}$ was 0.45 following 200 repetitions of the sampling, training, and testing sets. It can be seen that the model ensemble outperforms every single model.

### 2.9 sLORETA results

**FIGURE 8:**
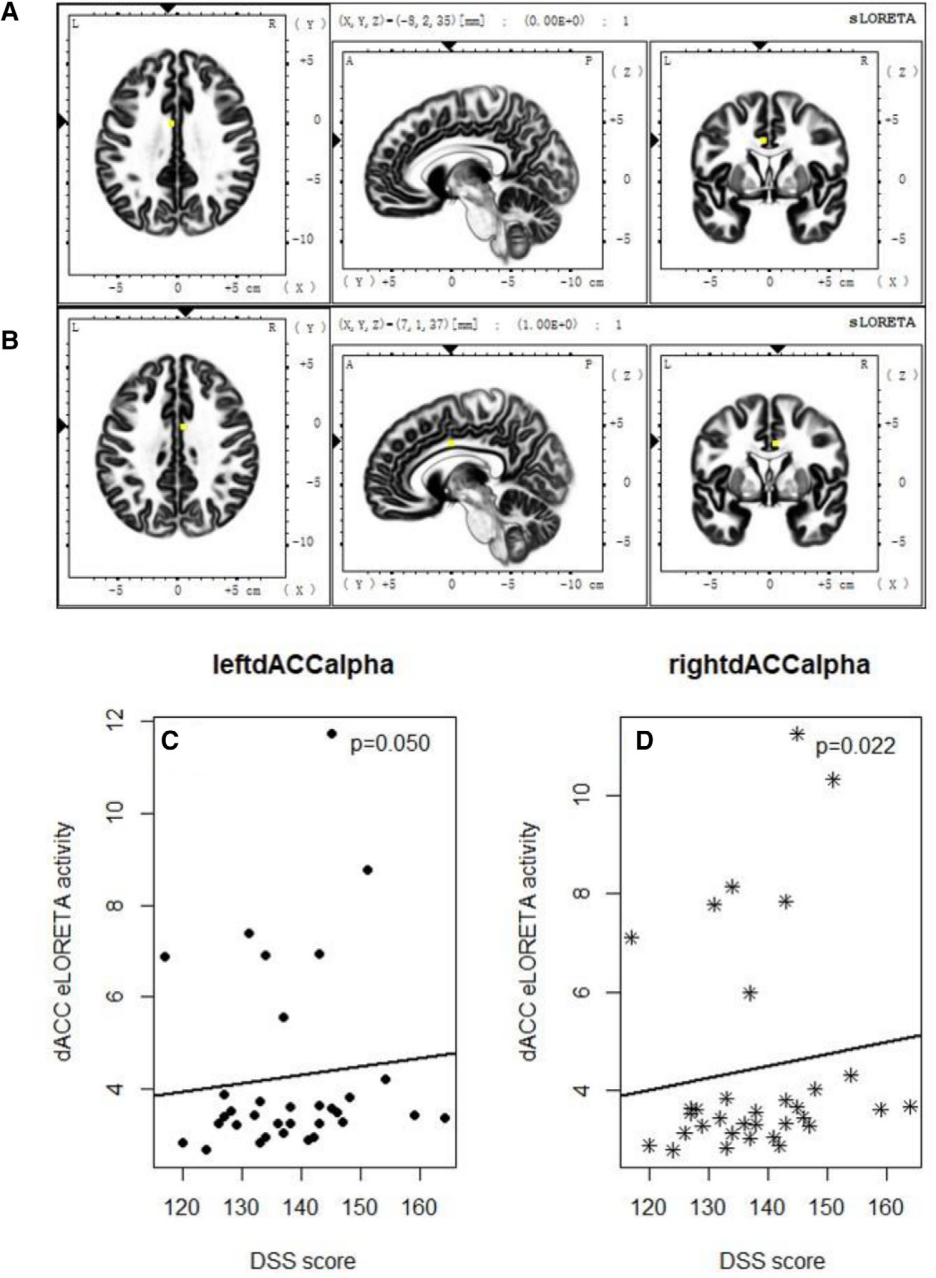
Dorsal anterior cingulate cortex (dACC) exact low‐resolution electromagnetic tomography (eLORETA) correlation with Dialectical Self Scale (DSS) scores. (a) and (b) eLORETA localization of the left and right dACC (a cortical midline structure) based on Montreal Neurological Institute coordinates from a previous study. (c) One‐tailed Spearman's correlations test between left dACC eLORETA alpha values and DSS scores. (d) One‐tailed Spearman's correlation test between the right dACC eLORETA alpha values and DSS scores

**FIGURE 9:**
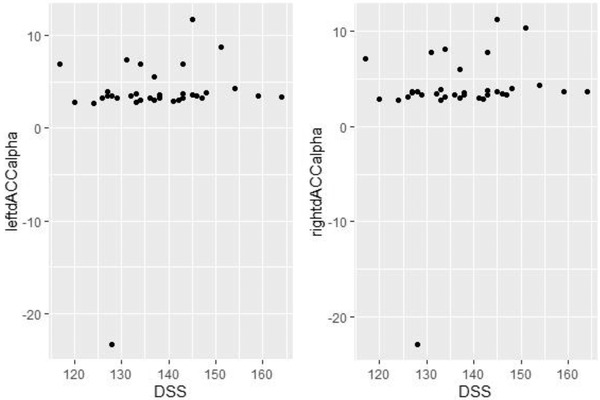
Scatterplot of dACC alpha value and DSS scores

We selected an area of interest from the existing literature (Damasio, 1996; De Ridder et al., 2011; Song et al., 2014). The two regions were the right and left dACC (Figure 8a and 8b). The scatterplot of dACC and DSS scores is displayed in Figure 9. As Figure 9 shows, there are two extreme left‐dACC and right‐dACC values (occurred at the left bottom of the plot), which may have a great influence on the Pearson correlation results. Thus, we perform the Spearman's correlation test, which is more robust in such an extreme value condition. Based on the results in Section 3.3.4, Spearman's correlation tests were performed between the DSS score and left dACC alpha wave and the right dACC alpha wave. In one‐tailed Spearman's correlation tests, the correlation values for the (left dACC and right dACC) eLORETA data were as follows: left dACC alpha, *r* = 0.287, *p* = 0.050; right dACC alpha, *r* = 0.346, *p* = 0.022 (Figure 8c and 8d), while other frequency bands did not significantly correlate with DSS. The *p* value of right dACC alpha passed Bonferroni correction at a significance level of 0.05.

The homogeneity test and Anderson and Darling (1954) against the normality of data were performed. The *p* value of homogeneity test was 0.095, indicating no significant difference of dACC variance in the two areas. The *p* value of the normality of the left‐dACC alpha value, right‐dACC alpha value, and DSS score were <0.001, <0.001, and 0.889, respectively. The strong evidence to reject the normality of dACC value may due to their extreme values (as shown in Figure 9). Therefore, the Spearman's correlation test was considered to be more appropriate in this study.

We also analyzed the precuneus, one of the areas with the lowest spontaneous activity (Coito et al., 2019) as a control. The precuneus is rarely activated in the resting state of EEG, so one could expect that it is not related to dialectical thinking in the resting‐EEG. The bilateral precuneus was located based on Montreal Neurological Institute (MNI) coordinates (left precuneus −12, −65, 50; right precuneus 12, −65, 50)(Kraft et al., 2015). Spearman's correlation tests were performed between the DSS score and left/right precuneus alpha wave. In one‐tailed Spearman's correlation tests, the correlation values for the (left precuneus and right precuneus) eLORETA data were as follows: left precuneus alpha, *r* = 0.037, *p* = 0.417; right precuneus alpha, *r* = −0.062, *p* = 0.364. No significant correlation was found between the alpha value of standardized low‐resolution electromagnetic tomography activity in the left/right precuneus and dialectical self‐scale score.

## 3 DISCUSSION

Our results show that the whole EEG series is not a good predictor of DSS, suggesting that a more precise analysis of data segments is required. Among the segmented periods, periods 4−6 at the beginning of data recording demonstrated good predictive value. Our interpretation of this result is that during these periods, the participants were still new to the experimental environment and were gradually adapting to it, and dispositional dialectical thinking was especially exerting its effect during the disengagement from external stimuli. However, the current resting‐state design limits our ability to finely delineate the exact events happened during the beginning phase. Future studies might directly examine the link between dispositional dialectical thinking and EEG signals when there is an overt task.

Among the machine learning methods, our results show that LASSO was the best in this study. LASSO is a linear model with shrinkage. It reduces the predicted variance while sacrificing a little bias so that the total prediction error is smaller (Tibshirani, 1996). The linear relationship is a simple assumption between the response and predictors, which makes it widely used in regression analysis. Because our sample size was rather small, we were more inclined to use a simple model in our problem. On the other hand, the number of feature dimensions after the FPCA procedure was approximately 10 for most ${\left\{w,p,l\right\}}_{1\leq w\leq 4,0\leq p\leq 84,1\leq l\leq 63}$ settings, which is slightly high relative to our sample size of 23 in the training set. While LASSO also serves as a method of feature selection due to its sparse estimation result (Tibshirani, 1996), it is reasonable to expect it to behave better after further dimension reduction in our small sample problem. In contrast, the other four machine learning methods’ (SVR, GBDT, KNN, and RF) prediction accuracy may be affected by some FPCA scores irrelevant to DSS score without feature subset selection.

While 4 × 84 × 63 = 21,168 ${\bar{R}}^{2}$ values were calculated for each machine learning method in segmental EEG analysis, one needs to be careful regarding results of ${\bar{R}}^{2}$ > 0 because false positive results occur during a large number of trials. Thus, whether these results are simply caused by random chance needs to be determined. The randomness test result rejects the null hypothesis for the identical individually independent distribution of $R_{w,p,l,m}^{2}$ for the LASSO method, which provides us more confidence in the relationship between useful predictors and DSS score. The test results show that there is a significant difference between the alpha wave and other three waves, while the difference among the other three waves was not significant enough to distinguish them. However, this conclusion should be made cautiously. The test statistic's distribution relies on the assumption of the identical individually independent distribution of signals from different periods and electrodes under each wave, which may not be true in real‐life situations. Thus, rejecting this hypothesis does not make much sense in some cases. On the other hand, the true relationship between different periods and electrodes are too complex to characterize, not to mention to consider in a hypothesis test. However, this test result does provide some evidence regarding the predictive ability of the alpha wave despite these limitations.

Furthermore, a model ensemble was used to strengthen the model's predictive ability. The model ensemble is a technique combining different models to achieve a better accuracy than any of its constituent models (Opitz & Maclin, 1999). In our case, the model ensemble achieved an average ${\bar{R}}^{2}$ of 0.45, while the single model's highest ${\bar{R}}^{2}$ was 0.376. This is due to a reduction in the error variance by averaging each single model's output. The performance of the model ensemble was affected by the correlation of its constituent models. Generally speaking, the more independence among the constituent models, the better the model ensemble will perform (Goodfellow et al., 2016). Because our constituent models were based on electrodes FC1, FCz, Fz, FC3, Cz, and AFz separately, they are not expected to share much dependence, which accounts for the increased prediction accuracy of our model ensemble. To compare with other relevant studies, Al Zoubi et al. (2018) build a model to predict age from EEG signal and achieve best $R^{2}$ = 0.37 (the number of subjects = 500) and Zhang et al. (2020) use EEG to predict the working memory and the model's $R^{2}$ = 0.72 (the number of subjects = 145). Considering the fact that only 30 subjects are used in this study, we think our model's performance is brilliant with $R^{2}$ = 0.45.

Finally, in the model, the electrodes that could predict the best DSS results were basically consistent with the results of the literature review, primarily measuring signals from the dACC and DLPFC. Consequently, an sLORETA source‐analysis approach was used, which was designed to estimate the location and activity of the neural generators that cause EEG activity in the scalp. We explored the correlation between DSS and cortical sources of resting cortical EEG rhythms (delta [1−4 Hz], theta [4−7 Hz], alpha [7−13 Hz], beta [13−30 Hz]). In the present study, we observed a positive correlation between right dACC resting alpha sources and DSS scores. There are several interpretations for this result (Sadaghiani & Kleinschmidt, 2016). First, alpha oscillations are associated with the inhibition of neural activity, a process that corresponds cognitively to the internal maintenance of tonic alertness, usually occurring during brain processes not directly related to tasks, similarly, the resting state EEG was used in this study. Second, alpha oscillations are associated with the cognitive function of selective attention, that is, the associated feature selection process takes precedence over other processes from top to bottom. Easterners with a higher degree of dialectical thinking pay more attention to relational situations than westerners(English & Chen, 2007) Third, alpha oscillations can also achieve rapidly changing long‐distance cortical coordination, which can be thought of as phase adaptive control, including the regulation of working memory. Given the dominant cultural norms in East Asia (i.e., ingroup harmony and collective agency), these strategies play a functionally adaptive role in everyday control exertion(Park et al., 2018). And alpha waves can be well identified using a data‐driven approach (Tenke & Kayser, 2015; Tenke et al., 2017). This showed that only the peculiar topography and frequency of cortical resting EEG sources were able to roughly discriminate between dialectic and nondialectical. These results are in line with previous findings suggesting that dACC alpha rhythms are one of the physiological mechanisms by which the associative dACC modulates conflict processing (Nakao et al., 2013; Strauss et al., 2012).

The current study has several limitations. First, the disadvantage of EEG is its spatial resolution. The 64 electrodes can only map a limited area of activity, and 256‐electrode set‐ups have a significant spatial resolution improvement over their 64‐electrode equivalents (Luu et al., 2001; Wu et al., 2014). Second, on this basis, there is controversy regarding whether spatial localization truly reflects changes in specific brain regions, which is worth investigating. In the future, using MRI and magnetoencephalography to investigate spatial changes in the brain will be a worthy research direction. Third, there are some hyperparameters in these five machine learning methods (e.g., regularization coefficient in LASSO model, number of nearest neighbors in KNN, etc.) that can be tuned to improve model accuracy. Because we included many {*w*,*p*,*l*} settings, it was impractical to tune these parameters individually. We decided upon these hyperparameters based on our empirical experience and successfully obtained approximate results. Since there has been no prior research on the prediction of DSS scores based on EEG data, our results can serve as a reference for a more precise study in the future.

## 4 CONCLUSION

We investigated the brain's spontaneous activity over time using resting EEG and linked it to dialectical thinking. There was a significant positive correlation between the alpha wave of sLORETA activity and DSS score in the right dACC brain region. Together with sLORETA analysis, our machine learning results show that LASSO is the best machine learning method, and the alpha wave is the best predictor of DSS score in this study. With data‐driven selected electrodes (FC1, FCz, Fz, FC3, Cz, AFz), the deterministic coefficient of the prediction model in the test set achieved an average of 0.45 among 200 repetitions. In summary, the prefrontal and midline alpha oscillations of resting EEG are good predictors of the dialectical thinking score, possibly reflecting these brain structures’ involvement in dialectical thinking.

## CONFLICT OF INTEREST

All other authors declare no conflicts of interest.

### PEER REVIEW

The peer review history for this article is available at https://publons.com/publon/10.1002/brb3.2327.

## Data Availability

The data and code of this study will be available on request to the corresponding author.

## APPENDIX A PROCEDURE OF ESTIMATING FUNCTIONAL PRINCIPAL COMPONENT SCORE

Consider a dataset consisting of *n* subjects with each subject taking a measurement every equidistant period and the total number of measurements per subject is N. The measured quantity, denoted as Y, can be any index, like notes the voltage, current, etc. We denote the dataset as ${\left\{Y_{ij}\right\}}_{1\leq i\leq n,1\leq j\leq N}$, where i denotes the *i*th subject and j denotes the *j*th measurement. Without loss of generality, assume the time interval between two measurements is $\frac{1}{N}$. We build our functional model

(A.1) $$Y_{i}\;\left(t\right)=\eta_{i}\;\left(t\right)+\varepsilon_{i}\left(t\right),\;t\in \left[0,1\right],$$

where $\eta_{i}\left(t\right)$ is the infeasible underlying time‐varying process of the subject *i*, which is the realization of stochastic process {η(t) *, t* ∈ [0,1]} with $E\int_{0}^{1}\eta^{2}\left(t\right)dt<\infty$. $\varepsilon_{i}\left(\cdot \right)$ is the error term of the *i*th subject. The observed data is the discretized realization of this model. Denote the covariance function of η(·) as$\;G\;\left(t,t^{\prime }\right)=\;Cov\left(\eta \left(t\right),\eta \left(t^{\prime }\right)\right)$. The classical functional analysis theory assures that there exist series of values $\lambda_{1}\geq \lambda_{2}\geq \ldots \geq 0$ with $\sum_{k\;=\;1}^{\infty }\lambda_{k}<\infty$ and series of orthonormal function basis ${\left\{\psi_{k}\left(\cdot \right)\right\}}_{k\geq 1}$ such that $G\;\left(t,t^{\prime }\right)=\sum_{k=1}^{\infty }\lambda_{k}\psi_{k}\left(t\right)\psi_{k}\left(t{}^{\prime }\right)\;$, $\int G\left(t,t^{\prime }\right)\psi_{k}\left(t^{\prime }\right)dt^{\prime }=\;\lambda_{k}\psi_{k}\left(t\right)$, where ${\left\{\lambda_{k}\left(\cdot \right)\right\}}_{k\geq 1}$ is called eigenvalue and $\phi_{k}\;\left(\cdot \right)=\sqrt{\lambda_{k}}\;\psi_{k}\left(\cdot \right)$ is called rescaled eigenfunctions of the covariance function$\;G(t,t{}^{\prime })$. Furthermore, we represent model 1 as the well known Karhunen‐Loève $L_{2}$ form:

(A.2) $$Y_{i}\;\left(t\right)=\;m\left(t\right)+\sum_{k\;=\;1}^{\infty }\xi_{ik}\phi_{k}\left(t\right)+\varepsilon_{i}\left(t\right),\;0\leq t\leq 1\;,$$

where m(·) is the mean function among all the subjects and $\eta_{i}\;\left(t\right)=\;m\left(t\right)+\sum_{k\;=\;1}^{\infty }\xi_{ik}\phi_{k}\left(t\right)$.

The FPC (functional principal component) score ${\left\{\xi_{ik}\right\}}_{1\leq i\leq n,k\geq 1}$is series of uncorrelated random variable with mean zero and variance 1 representing the complex structure behind the data. Since the observed data is discrete, the practical model is

(A.3) $$Y_{i}\;\left(\frac{j}{N}\right)=\;m\left(\frac{j}{N}\right)+\sum_{k\;=\;1}^{\infty }\xi_{ik}\phi_{k}\left(\frac{j}{N}\right)+\varepsilon_{i}\left(\frac{j}{N}\right),\;1\leq j\leq N.$$

The procedure of estimating FPC score is decomposed into following steps.

### A.1 B‐spline estimation

The first step is approximating the original signal by B‐spline basis functions. B‐spline basis has been widely used in function approximation and nonparametric statistics. The B‐spline approximation is quite fast since the calculation can be done by simple matrix operation. With predetermined hyperparameter $J_{s}$, interval [0,1] is segmented into $J_{s}$ equal length subinterval, denotes as ${\left\{I_{l}\right\}}_{l\;=\;0}^{J_{s}}$, with interior knots ${\left\{t_{l}\right\}}_{l\;=\;1}^{J_{s}},$ where $t_{l}=\frac{l}{J_{s}+1}\;$. For any positive integer *p*, define some auxiliary knots as$\;t_{1-p}\;=\;\cdot \cdot \cdot \;=\;t_{0}\;=\;0$ and $t_{J_{s}+1}\;=\;\cdot \cdot \cdot \;=\;t_{J_{s}+p}\;=\;1$. Following the notation in De Boor (1978), denote the *p*th order B‐spline basis functions as $B_{l,p}\left(x\right),1\;\leq \;l\;\leq \;J_{s}+p\}$. Then the polynomial spline space of order p can be denoted as $S^{\left(p-2\right)}=\left\{\sum_{l=1}^{J_{s}+p}\lambda_{l,p}B_{l,p}\left(x\right)|\lambda_{l,p}\in R,x\in \left[0,1\right]\right\}\;$. The idea is to approximate $\eta_{i}\left(\cdot \right)$ by the function of $S^{\left(p-2\right)}$. Denote $Y_{i}={\left[Y_{i1},\ldots \ldots ,Y_{iN}\right]}^{T}\;$, then the approximated trajectory of subject i is obtained by

(A.4) $${\hat{\eta }}_{i}\;\left(\cdot \right)=\;argmin_{g\in S^{\left(p-2\right)}}\sum_{j\;=\;1}^{N}{\left(Y_{ij}-g\left(\frac{j}{N}\right)\right)}^{2}$$

Since the $S^{\left(p-2\right)}$ space is linear combination of the spline basis, ${\hat{\eta }}_{i}\left(\cdot \right)$ can be denoted as${\hat{\eta }}_{i}\;\left(\cdot \right)=\;B{\left(\cdot \right)}^{T}{\hat{b}}_{i},$ where B(·) and ${\hat{b}}_{i}$ are defined as $B\;\left(\cdot \right)={\left[B_{1,p}\left(\cdot \right),B_{2,p}\left(\cdot \right),\ldots \ldots ,B_{Js+p,p}\left(\cdot \right)\right]}^{T}\;$,$\;\;{\hat{b}}_{i}={\left[{\hat{b}}_{i,1},\;\ldots \ldots ,{\hat{b}}_{J_{s}+p,1}\right]}^{T}\;$. Furthermore, define a matrix **B** $={\left(\begin{array}{ccc} B_{1,p}\left(\frac{1}{N}\right) & \ldots & B_{J_{s}+p,p}\left(\frac{1}{N}\right)\\ \vdots & \ddots & \vdots \\ B_{1,p}\left(\frac{N}{N}\right) & \cdots & B_{J_{s}+p,p}\left(\frac{N}{N}\right) \end{array}\right)}_{N\times (J_{s}+p)}\;,$ then it can be shown that $b_{i}$ estimated from equation 4 is ${\hat{b}}_{i}={\left(B^{T}B\right)}^{-1}\;B^{T}Y_{i}$. The spline approximation function of $\eta_{i}\left(\cdot \right)$ is ${\hat{\eta }}_{i}\;\left(\cdot \right)=\;B{\left(\cdot \right)}^{T}{\left(B^{T}B\right)}^{-1}B^{T}Y_{i}$.

### A.2 Estimation of covariance function $G(t,t^{\prime })$

The definition of $G(t,t{}^{\prime })$ is $G\;\left(t,t^{\prime }\right)=\;Cov\;\left(\eta \left(t\right),\eta \left(t^{\prime }\right)\right)=\;E\left(\eta (t)-m(t)\right)\left(\eta \left(t^{\prime }\right)-m\left(t^{\prime }\right)\right)$, where m(·) is first estimated by $\hat{m}\;\left(\cdot \right)=n^{-1}\;\sum_{i\;=\;1}^{n}{\hat{\eta }}_{i}\left(\cdot \right)$. Then the $G(t,t{}^{\prime })$ can be estimated by $\hat{G}\;\left(t,t^{\prime }\right)=n^{-1}\;\sum_{i\;=\;1}^{n}\left({\hat{\eta }}_{i}\left(t\right)-\hat{m}\left(t\right)\right)\left(\hat{\eta }\left(t^{\prime }\right)-\hat{m}\left(t^{\prime }\right)\right)$. Since the ${\hat{\eta }}_{i}\left(t\right)$ and $\hat{m}\left(t\right)$ are both spline functions, it can be shown that $\hat{G}\;\left(t,t^{\prime }\right)=\sum_{j=1}^{J_{s}+p}\sum_{j^{\prime }=1}^{J_{s}+p}{\hat{\alpha }}_{j,j^{\prime }}B_{j,p}\left(t\right)B_{j^{\prime },p}\left(t^{\prime }\right)\;$, where ${\hat{\alpha }}_{j,j^{\prime }}=n^{-1}\;\sum_{i\;=\;1}^{n}\left({\hat{b}}_{ij}-n^{-1}\sum_{i\;=\;1}^{n}{\hat{b}}_{ij}\right)\left({\hat{b}}_{ij^{\prime }}-n^{-1}\sum_{i\;=\;1}^{n}{\hat{b}}_{ij^{\prime }}\right)$ and ${\hat{b}}_{ij}$ denotes the *j*th element of ${\hat{b}}_{i}$. Furthermore, denote the matrix formed by ${\left\{{\hat{\alpha }}_{j,j^{\prime }}\right\}}_{1\leq j\leq J_{s}+p,1\leq j^{\prime }\leq J_{s}+p}$as

$$\;\hat{\alpha }={\left(\begin{array}{ccc} {\hat{\alpha }}_{1,1} & \ldots & {\hat{\alpha }}_{1,\;J_{s}+p}\\ \vdots & \ddots & \vdots \\ {\hat{\alpha }}_{J_{s}+p,1} & \cdots & {\hat{\alpha }}_{J_{s}+p,\;J_{s}+p} \end{array}\right)}_{\left(J_{s}+p\right)\times \left(J_{s}+p\right)}\;.$$

### A.3 Estimation of FPC score, eigenvector, and eigenvalues

For any positive integer *k*, the *k*th eigenvalue $\lambda_{k}$ and eigenfunction corresponding to it satisfies equation

(A.5) $$\;\int G\left(t,t^{\prime }\right)\psi_{k}\left(t^{\prime }\right)dt^{\prime }=\;\lambda_{k}\psi_{k}\left(t\right).$$

We use $\hat{G}\left(t,t^{\prime }\right)$ to replace $G(t,t{}^{\prime })$ and approximate $\psi_{k}\left(\cdot \right)$ by spline functions, denoted as ${\hat{\psi }}_{k}\;\left(\cdot \right)=\;B{\left(\cdot \right)}^{T}{\hat{\gamma }}_{k}$, where ${\hat{\gamma }}_{k}={\left[{\hat{\gamma }}_{k,1},\ldots ,{\hat{\gamma }}_{k,J_{s}+p}\right]}^{T}\;$. Denote spline basis functions’ inner product matrix as

$$H\;={\left(\begin{array}{ccc} \int B_{1,p}\left(t\right)B_{1,p}\left(t\right)dt & \ldots & \int B_{1,p}\left(t\right)B_{J_{s}+p,p}\left(t\right)dt\\ \vdots & \ddots & \vdots \\ \int B_{J_{s}+p,p}\left(t\right)B_{1,p}\left(t\right)dt & \cdots & \int B_{J_{s}+p,p}\left(t\right)B_{J_{s}+p,p}\left(t\right)dt \end{array}\right)}_{\left(J_{s}+p\right)\times \left(J_{s}+p\right)}\;.$$

Then it can be shown by simple algebra that the equation A.5 is equivalent to ${\hat{\lambda }}_{k}\;{\hat{\gamma }}_{k}=\hat{\alpha }\;H{\hat{\gamma }}_{k}$. The matrix H can further be decomposed as $H\;=\;DD^{T}$ by Cholesky decomposition, which electrodes to the equation $D^{T}\hat{\alpha }DD^{T}\;{\hat{\gamma }}_{k}=\;{\hat{\lambda }}_{k}D^{T}{\hat{\gamma }}_{k}$. Then the real value ${\hat{\lambda }}_{k}$ and the vector $D^{T}{\hat{\gamma }}_{k}$ can be regarded as eigenvalue and eigenvector of the matrix $D^{T}\hat{\alpha }D$ and ${\hat{\lambda }}_{k}$ and ${\hat{\psi }}_{k}\left(\cdot \right)$ can be obtained therefore.

Notice that the model condition implies the equation $\int \left(\eta_{i}\left(t\right)-m\left(t\right)\right)\phi_{k}\left(t\right)dt\;=\lambda_{k}\;\xi_{ik}$. Replace with the estimated term, one gets $\int \left({\hat{\eta }}_{i}\left(t\right)-\hat{m}\left(t\right)\right){\hat{\phi }}_{k}\left(t\right)dt\;={\hat{\lambda }}_{k}\;{\hat{\xi }}_{ik}$. Since ${\hat{\eta }}_{i}\left(\cdot \right)$, $\hat{m}\left(\cdot \right),$ and ${\hat{\phi }}_{k}\left(\cdot \right)$ are spline functions, after some algebraic operations, the FPC score estimation are obtained by ${\hat{\xi }}_{ik}={\hat{\lambda }}_{k}^{-1/2}\;\mu_{i}^{T}H{\hat{\gamma }}_{k}$, where

$$\mu_{i}={\left(B^{\prime }B\right)}^{-1}\;B^{T}\;\left(Y_{i}-n^{-1}\sum_{i\;=\;1}^{n}Y_{i}\right)={\hat{b}}_{i}\;-n^{-1}\sum_{i\;=\;1}^{n}{\hat{b}}_{i}.$$

Finally, the number of FPC score is chosen by the rule of thumb criterion:

$$\kappa \;=\mathrm{argmi}n_{1\leq q\leq J_{s}+p}\;\left\{\left(\sum_{k\;=\;1}^{q}\;{\hat{\lambda }}_{k}/\sum_{k\;=\;1}^{J_{s}+p}\;{\hat{\lambda }}_{k}\right)>0.95\right\}.$$

The first κ FPC score are selected, by which one is assumed to have obtained enough information from the original signal.
